# Comparative study of total-body PET and PET/MR in the diagnosis of liver metastases

**DOI:** 10.3389/fonc.2025.1519107

**Published:** 2025-05-14

**Authors:** Cao Junxia, Xuanyuan Yirui, You Yang, Zhang Weifeng, Xuan Ang

**Affiliations:** ^1^ Department of Medical Imaging, Henan Provincial People’s Hospital, People’s Hospital of Zhengzhou University, Zhengzhou, China; ^2^ West China School of Medicine, Sichuan University, Chengdu, China; ^3^ PET/CT Center, Department of Nuclear Medicine, Henan Provincial People’s Hospital, People’s Hospital of Zhengzhou University, Zhengzhou, China

**Keywords:** positron emission tomography/computed tomography, magnetic resonance imaging, liver metastasis, PET-CT, PET-MRI

## Abstract

**Objective:**

To compare the diagnostic differences between total-body PET/CT (positron emission tomography/computed tomography) and PET/MR (positron emission tomography/magnetic resonance) in detecting liver metastases.

**Methods:**

The study analyzed data from patients with malignancies who underwent both conventional total-body PET/CT and liver PET/MR imaging between June 2020 and December 2020. A total of 20 patients with confirmed liver metastases were included, 9 of whom also underwent 2-hour delayed imaging of the liver. Paired t-tests were used to compare the signal-to-noise ratio (SNR) and tissue-to-background ratio (T/B) between PET/MR and conventional total-body PET/CT. Wilcoxon non-parametric tests were used to compare the standardized uptake value maximum (SUVmax) between the two imaging modalities. The McNemar test was employed to assess diagnostic performance differences between PET/MR and conventional total-body PET/CT, as well as between PET/MR and 2-hour delayed total-body PET/CT.

**Results:**

A total of 20 patients with confirmed liver metastases were included, with 39 suspicious lesions identified, and 27 lesions confirmed as liver metastases through biopsy or follow-up. The sensitivity of total-body PET/CT was 66.7% (18/27), while PET/MR had a sensitivity of 96.3% (26/27). The specificity of total-body PET/CT was 83.3% (10/12), and PET/MR had a specificity of 91.7% (11/12). The McNemar test revealed a significant difference in diagnostic performance between the two modalities, with PET/MR outperforming conventional total-body PET/CT (p=0.016). In 9 patients who underwent 2-hour delayed total-body PET/CT, 10 suspicious lesions were identified, 8 of which were confirmed as liver metastases. The sensitivity of delayed total-body PET/CT was 75% (6/8), and PET/MR had a sensitivity of 87.5% (7/8). Both modalities had a specificity of 50% (1/2). The McNemar test for delayed imaging showed no statistically significant difference (p=1). Wilcoxon non-parametric testing showed that the SUVmax of total-body PET/CT was significantly higher than that of PET/MR (Z=-2.355, p=0.019). Paired t-tests indicated no significant differences in SNR (t=-1.565, p=0.156) and T/B ratio (t=-1.689, p=0.115) between the two modalities.

**Conclusion:**

Total-body PET/CT demonstrated higher detector sensitivity compared to PET/MR. However, PET/MR showed superior diagnostic performance for detecting liver metastases. The delayed 2-hour PET/CT imaging could partially compensate for the lower diagnostic efficiency of conventional PET/CT compared to PET/MR.

## 1 Introduction

The liver is the most common site for metastasis of malignant tumors, with liver metastases accounting for approximately 25% of all malignancies (1). The presence of liver metastases in cancer patients indicates a poor prognosis and a higher mortality risk. Early detection of liver metastases and intervention are of great clinical significance. Imaging examinations provide reliable information for the early screening and therapeutic assessment of liver metastases. The advent of ^18^F-fluorodeoxyglucose positron emission tomography/computed tomography (^18^F-FDG PET/CT) has been revolutionary, as it provides both anatomical and metabolic information of lesions. However, conventional PET/CTemploying non-diagnostic CT and the low soft tissue resolution of CT itself limit the detection of certain lesions (2). Total-body PET/CT was known to significantly improve the PET image quality due to its ultra-high sensitivity.2-hour delayed imaging, have been explored to enhance PET/CT diagnostic performance for liver metastases (3, 4). The emergence of PET/MR, which combines the multi-parametric imaging capabilities of magnetic resonance imaging (MRI) with the functional imaging of PET, compensates for the lack of anatomical resolution in PET/CT, significantly improving soft tissue lesion detection (5, 6). However, few studies have compared the diagnostic efficacy of conventional total-body PET/CT with PET/MR, and the differences between delayed total-body PET/CT and PET/MR in detecting liver metastases. This study summarizes the imaging characteristics of total-body PET/CT and PET/MR in 20 patients with liver metastases and compares their diagnostic efficacy, aiming to provide valuable clinical insights.

## 2 Materials and Methods

### 2.1 General information

The study analyzed data from 20 patients with malignancies who underwent total-body PET/CT and liver PET/MR at the PET/CT Center of Henan Provincial People’s Hospital between June 2020 and December 2020. The 20 patients (15 males, 5 females, mean age 56.64 ± 11.04 years) with 39 suspected lesions were included. In 39 suspected lesions, a total of 27 liver metastases were confirmed by biopsy or follow-up (primary tumors:Lung cancer 10 cases, including 7 adenocarcinomas, 2 squamous carcinomas, 1 small cell carcinoma, T1-T2, N0-3, M1 stage (liver metastasis only). 5 cases of colon cancer, selected T1-T3, N0 or N1, M1 (liver metastasis only). Gastric cancer 3 cases, choose T1-T3, N0 or N1, M1 (liver metastasis only). Breast cancer 2 cases, choose T1-T3, N0-N3, M1 (liver metastasis only)).

### 2.2 Methods

#### 2.2.1 ^18^F-FDG total-body PET/CT imaging

The imaging equipment used was the United Imaging Healthcare uEXPLORER total-body PET/CT scanner. The ^18^F-FDG was synthesized using a GE Mini Trace medical cyclotron (from the USA) and an FDG automatic synthesis device (provided by Beijing PET Co., Ltd.) with a radiochemical purity of over 95%. Prior to the examination, patients fasted for more than 6 hours and blood glucose levels were measured via fingertip blood tests, ensuring fasting blood glucose levels were controlled below 6.1 mmol/L. Intravenous injection of ^18^F-FDG was administered at a dose of 5.55 MBq/kg. After 40 minutes of resting with eyes closed in a temperature-appropriate environment, standard imaging was conducted. Nine patients underwent delayed imaging 2 hours later. The patient’s head was fixed during imaging, and total-body image acquisition was performed using a CT transmission scan followed by a PET emission scan. Patients were positioned supine with their hands behind their heads, breathing calmly, and the head was scanned first.

The scan range for standard imaging extended from the top of the skull to the toes (strictly total-body scanning), while for the 2-hour delayed imaging, the scan focused on the liver. The CT scan parameters were as follows: for the torso scan, tube voltage was set to 120 kV, tube current at 150 mAs, pitch of 0.9625, scan field of view (FOV) was 50.0 cm, reconstruction slice thickness was 3 mm, interslice spacing was 1.5 mm and the CT reconstruction matrix was 512×512. The PET acquisition parameters were as follows: data was collected in 3D mode for 5 minutes per bed position (one bed position total), matrix size of 256 × 256, PET reconstruction slice thickness was 1.443 mm and PET images were reconstructed using the iterative method. Attenuation correction for the total-body PET images was performed using attenuation correction parameters derived from CT.

#### 2.2.2 ^18^F-FDG PET/MR imaging

The imaging equipment used was the United Imaging Healthcare uPMR 790 PET/MR scanner. After Total-body PET/CT, the patients underwent liver PET/MR imaging while their body positions remained fixed. The scan sequences included both PET and MR imaging, performed simultaneously. The PET scan parameters were: resolution of 192×192, FOV of 50 cm, slice thickness of 2 mm and a total scan time of 25 minutes. The MR scan parameters were as follows: T1 parameters—slice thickness of 4.5 mm, FOV of 40 cm, repetition time (TR) of 4.28 ms, echo time (TE) of 1.14 ms; T2 fat-suppressed parameters—slice thickness of 6.0 mm, 28 slices, interslice spacing of 20 mm, FOV of 38 cm, TR of 3105 ms, TE of 90.2 ms; DWI parameters—slice thickness of 6.0 mm, 28 slices, interslice spacing of 20 mm, FOV of 38 cm, TR of 4223 ms, TE of 69.8 ms, DWI (b = 50, 800 s/mm2).

#### 2.2.3 Data analysis

Two senior nuclear medicine specialists independently analyzed the PET/CT and PET/MR data. In cases of disagreement, a consensus was reached through discussion. The following data were statistically analyzed: Maximum standardized uptake value (SUVmax) of liver metastases on Total-body PET/CT and PET/MR scans. Average liver background SUV on Total-body PET/CT and PET/MR scans (measured across three non-lesional layers, averaged with standard deviation calculated). Metastatic lesions: 39 lesions in total, including those detected by both Total-body PET/CT and PET/MR, those detected by PET/CT but not by PET/MR and those detected by PET/MR but not by PET/CT. In cases of delayed Total-body PET/CT imaging (10 lesions), comparison of the number of metastatic lesions detected by both PET/CT and PET/MR, by PET/CT but not PET/MR and by PET/MR but not PET/CT. The signal-to-noise ratio (SNR) for Total-body PET/CT and PET/MR was calculated as follows: the liver background SUV/standard deviation was used. The tissue-to-background ratio (T/B) for Total-body PET/CT and PET/MR was also calculated as the liver metastasis SUVmax/liver background average SUV.

#### 2.2.4 Statistical analysis

Data were processed using SPSS 23.0 software. Paired sample t-tests were used to compare the signal-to-noise ratios (SNR) and tissue-to-background ratios (T/B) between PET/MR and Total-body PET/CT (as the data conformed to normal distribution). The Wilcoxon non-parametric test was used to compare SUVmax between PET/MR and Total-body PET/CT (as the SUVmax data for PET/MR did not conform to normal distribution, p = 0.002). The McNemar test was used to compare the diagnostic performance of Total-body PET/CT and PET/MR, as well as between 2-hour delayed PET/CT and PET/MR. A p-value of less than 0.05 was considered statistically significant.

## 3 Results

**Figure 1:**
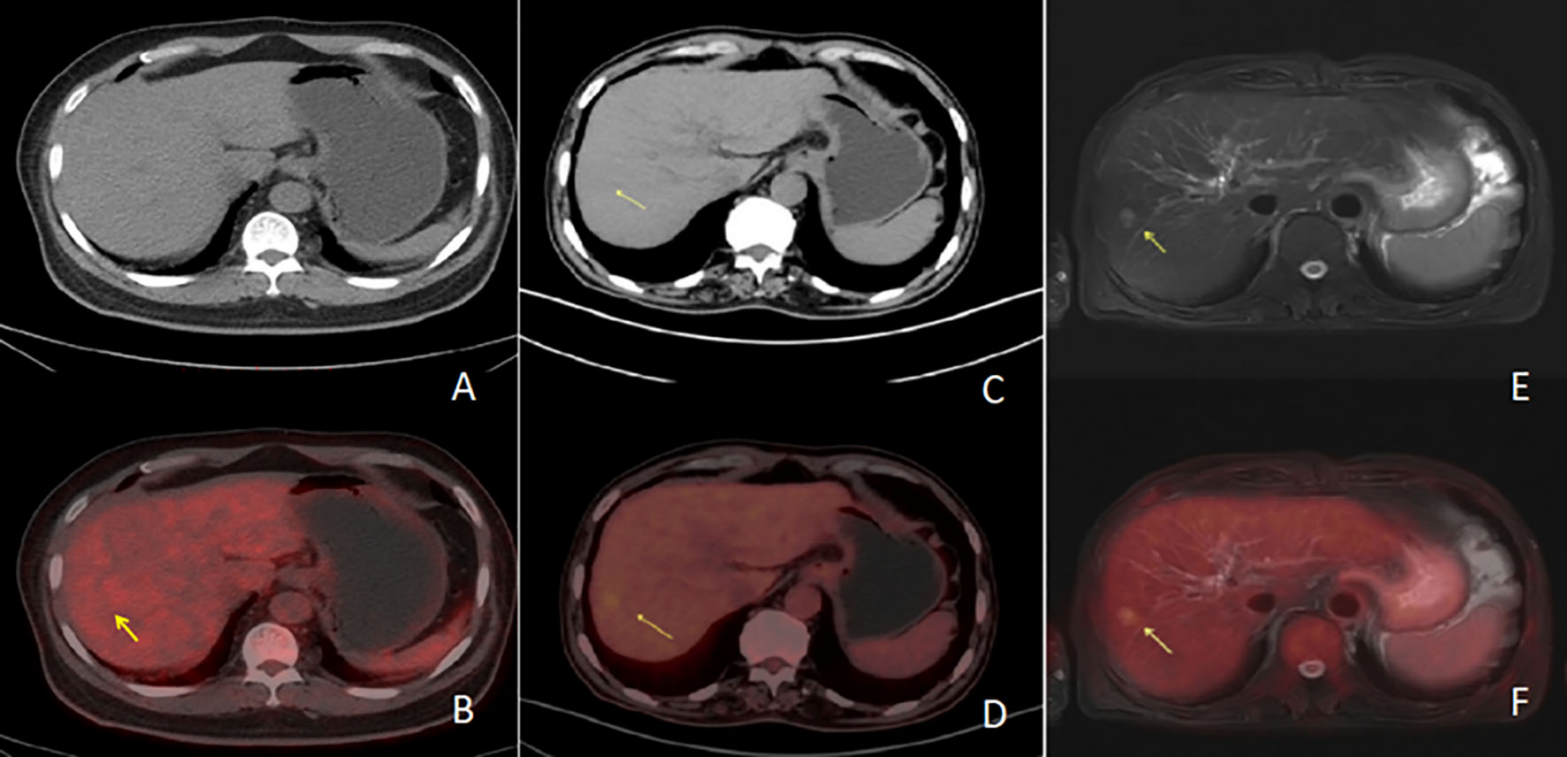
Row 1 **(A, B, E)** shows PET/CT images [**(A)** is a PET/CT CT image, **(B)** is a PET/CT fusion image, and **(E)** is a PET/CT PET image], and the second row **(C, D, F)** shows PET/MR images [**(C)** is a PET/MR MR image, **(D)** is a PET/MR fusion image, and **(F)** is a PET/MR PET image]. The CT image of the PET/CT lesions showed hypointensity with increased metabolism and a SUVmax of 6.4. In PET/MR images, T2WI lesions showed high signal with increased metabolism and a SUVmax of 3.7.

**Figure 2:**
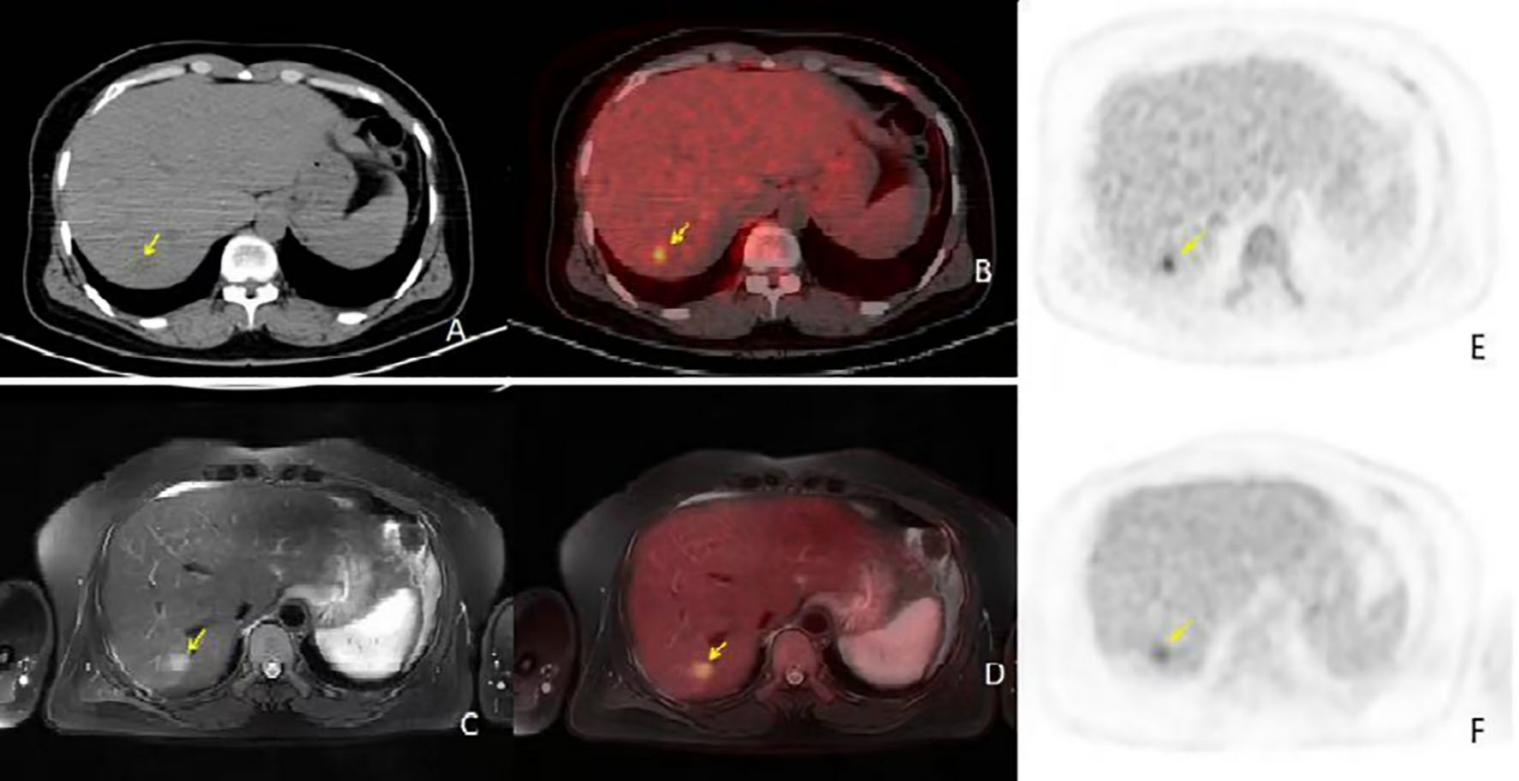
**(A, B)** are conventional PET/CT scan images [**(A)** is the CT image, **(B)** is the fusion image], **(C, D)** are PET/CT 2-hour delayed scan images [**(C)** is the CT image, **(D)** is the fusion image], and E and F are PET/MR images [**(E)** is the T2WI image, **(F)** is the fusion image]. The PET/CT lesion in the CT image shows isodensity, with no significant metabolic activity in the conventional scan. In the delayed scan, mild metabolic activity is seen, approaching the liver parenchyma, SUVmax 4.5. In the PET/MR image, the T2WI lesion shows high signal with mild metabolic activity, SUVmax 4.0. PET/MR provided a clearer diagnosis compared to PET/CT, and the PET/CT 2-hour delayed imaging offered a clearer diagnosis compared to conventional imaging.

Among the 20 patients with malignant tumors who underwent both Total-body PET/CT and liver PET/MR imaging, 15 were male and 5 were female, with an average age of 56.64 ± 11.04 years. All of 20 patients had liver metastases and a total of 39 suspected metastatic liver lesions were identified. Of these, 27 lesions were confirmed as liver metastases by follow-up or biopsy. The sensitivity of conventional Total-body PET/CT was 66.7% (18/27), while PET/MR had a sensitivity of 96.3% (26/27). The specificity of conventional Total-body PET/CT was 83.3% (10/12), and that of PET/MR was 91.7% (11/12). McNemar’s test showed significant differences between the two methods (n=39, p=0.016). Among the 9 patients who underwent both Total-body PET/CT and 2-hour delayed imaging, 10 lesions were identified, of which 8 were confirmed as liver metastases. The sensitivity of delayed Total-body PET/CT was 75% (6/8), while PET/MR had a sensitivity of 87.5% (7/8). The specificity of both delayed Total-body PET/CT and PET/MR was 50% (1/2). McNemar’s test for these 8 cases showed no statistically significant difference (p=1). The SUVmax values of Total-body PET/CT (8.01 ± 5.42) were significantly higher than those of PET/MR (6.38 ± 4.27), with a Z-value of -2.355 and a p-value of 0.019, indicating statistical significance. The signal-to-noise ratios (SNR) for Total-body PET/CT and PET/MR were 13.57 ± 9.24 and 19.53 ± 9.17, respectively (t=-1.565, p=0.156). The tissue-to-background ratios (T/B) were 3.25 ± 1.58 for Total-body PET/CT and 3.94 ± 1.98 for PET/MR (t=-1.689, p=0.115) with no statistically significant differences between the two (see **Table 1**). Representative images are shown in **Figures 1**, **2**. PET/MR did not detect one liver metastatic tumor lesion, located in the S7 segment of the liver, with a diameter of 4 mm, with no increase in FDG and DWI, and DWI was poorly displayed by respiratory artifacts.

**Table 1:** Comparative of Total-body PET/C and PET/MR in SUVmax、SNR、T/B and p value.

	Whole-Body PET/CT	PET/MR	T or Z	p
SUVmax	8.01 ± 5.42	5.1(3.8,7.1)^*^	Z=-2.355	p=0.019
SNR	13.57 ± 9.24	19.53 ± 9.17	t=-1.565	p=0.156
T/B	3.25 ± 1.58	3.94 ± 1.98	t=-1.689	p=0.115

*indicates that the data does not conform to the normal distribution, represented by M (P(25), P(75)), while the remaining data conforms to the normal distribution, represented by mean ± standard deviation. SNR: the signal-to-noise ratio; T/B: tissue-to-background ratio.

## 4 Discussion

PET is considered the most sensitive *in vivo* imaging technology for studying human physiology, molecular processes and metabolism. PET/CT imaging has been widely used for diagnosing and evaluating the prognosis of tumors, inflammation, and other functional diseases. However, due to the low soft tissue resolution of PET/CT, its application is somewhat limited in organs such as the nervous system, liver, kidneys, and prostate. The Total-body PET/CT is a new imaging device developed by United Imaging in China, which significantly improves the sensitivity of the PET detector, partially addressing the limitations of conventional PET/CT in terms of CT anatomical resolution.

PET/MR is an integrated imaging diagnostic device that combines positron emission tomography (PET) and magnetic resonance imaging (MRI), representing the forefront of functional and molecular imaging technology. PET/MR has the combined capabilities of both PET and MR, providing complementary advantages. Compared to CT, MR offers better soft tissue contrast, making it particularly suitable for detecting primary tumors and metastases in the nervous system, breast, liver, and other soft tissues. Many scholars, both domestically and internationally, believe that PET/MR has a significant advantage over conventional PET/CT in diagnosing liver metastases (7). Meanwhile, during the widespread use of PET/CT, researchers suggested that the metabolic peak of malignant tumors occurs approximately 2 hours after injection, making delayed imaging a potential method for improving diagnostic accuracy. Both Total-body PET/CT, 2-hour delayed PET/CT and PET/MR can increase lesion detection rates and assist in clinical diagnosis and treatment. However, there have been no reports comparing the diagnostic efficacy of these three modalities. This study aimed to compare the diagnostic performance of PET/MR with that of Total-body PET/CT and 2-hour delayed PET/CT in detecting liver metastases, providing guidance for clinical applications.

This study found that the SUVmax values for Total-body PET/CT were significantly higher than those for PET/MR. However, there were no statistically significant differences in the signal-to-noise ratio (SNR) or tissue-to-background ratio (T/B) between Total-body PET/CT and PET/MR. This result contradicts previous studies that reported higher SNR and T/B values for PET/MR (8–10). The higher SUVmax values for Total-body PET/CT suggest that Total-body PET/CT has higher system sensitivity, which refers to the ability of the detector to obtain counts under the same conditions. Its physical definition is the number of coincidence counts obtained per unit time and per unit radiation dose. A PET detector with higher sensitivity requires less time or a lower tracer activity to acquire an image of the same quality. Some researchers have pointed out that there are two ways to improve system sensitivity: increasing the axial field of view and improving time resolution (11). The axial field of view of the Total-body PET/CT by United Imaging is 2 meters, and with a data acquisition time of 5 minutes per bed position, it significantly enhances time resolution, leading to a substantial improvement in system resolution. This may explain why the SUVmax values of the same lesions are higher on Total-body PET/CT than on PET/MR. As system resolution improves, SNR and T/B ratios also increase, narrowing the differences between PET/CT and PET/MR. This could explain why, contrary to previous reports, there were no statistically significant differences between PET/CT and PET/MR in terms of SNR and T/B in this study.

Among the 39 suspected liver metastases, 27 lesions were confirmed as liver metastases by follow-up or biopsy. The sensitivity of Total-body PET/CT was 66.7% (18/27), and PET/MR had a sensitivity of 96.3% (26/27). The specificity of Total-body PET/CT was 83.3% (10/12), while PET/MR had a specificity of 91.7% (11/12). The results of the paired χ² test showed a statistically significant difference, indicating that PET/MR was able to detect more liver metastases, which is consistent with previous reports (7, 12–15). Upon retrospective analysis of cases where Total-body PET/CT missed lesions that were detected by PET/MR, it was found that the high system resolution of Total-body PET/CT increased the SUV values of normal liver tissue, making the lesion metabolism slightly higher than the surrounding tissue, leading to the lesion being easily overlooked visually and thus reducing specificity. PET/MR, with its advantage of combining multi-parametric imaging with metabolic imaging (lesions show long T1-weighted and T2-weighted signals, with restricted diffusion in DWI), greatly improved diagnostic efficiency. In summary, the results of this study demonstrate that PET/MR is superior to Total-body PET/CT in diagnosing liver metastases, particularly in lesion detection rates. This is closely related to the multi-parametric imaging capability of MR, while the Total-body PET/CT shows obvious optimization in terms of improved system resolution, compensating to some extent for the low soft tissue resolution of CT. The significant improvement in diagnostic efficiency by PET/MR is primarily attributed to its superior soft tissue resolution.

One case of liver metastases in our study that was not detected by PET/CT and PET/MR was found in our follow-up, which may be related to the small lesion or the location of a specific site, and the pathological type of the primary tumor may also have an impact on the secondary disease. Smaller liver metastases sometimes lack specificity on anatomical imaging, whereas in functional imaging, the lesion is sometimes isometabolic or hypometabolic due to the activity of the hepatic radiotracer or partial volumetric effects, which may lead to misdiagnosis (16), which also suggests that the larger the lesion, the higher the diagnostic power of PET/CT or PET/MR. Therefore, although PET/MR improves diagnostic efficacy, false negatives may still occur when the lesions are small. Although the diagnostic performance of PET/CT or PET/MR is related to the size of liver metastases, the diagnostic performance of liver metastases varies among different types of tumors. Brendle et al. reported PET/MR (MR/DWI/PET) without contrast enhancement showed a relatively lower sensitivity (71%), specificity (80%), as well as diagnostic accuracy (74%) for liver metastases in colorectal cancer. This was mainly because the data contained a relatively high percentage of mucinous tumors, which are known to be challenging for both DWI and PET evaluation (17).For the same type of tumor, the metabolic pattern of liver metastases may also be different, gastrointestinal stromal tumor (GIST) liver have different metabolic patterns (16).Through the above discussion, the diagnostic efficiency of liver metastases is affected by many factors, and the imaging characteristics of liver metastases of different types and stages of tumors should be explored in future experimental design.

Among the 9 patients who underwent both Total-body PET/CT and 2-hour delayed imaging, a total of 10 lesions were identified, 8 of which were confirmed as liver metastases. The sensitivity of delayed Total-body PET/CT was 75% (6/8), while PET/MR had a sensitivity of 87.5% (7/8). The specificity of both delayed Total-body PET/CT and PET/MR was 50% (1/2). McNemar’s test indicated no statistically significant difference between these two methods. Previous studies have generally shown that 2-hour delayed imaging can detect more primary and metastatic malignant lesions compared to conventional imaging. In this study, when comparing 2-hour delayed imaging with PET/MR, no statistically significant difference in diagnostic efficacy was found. This suggests that when PET/MR is not available in clinical practice, 2-hour delayed imaging can improve the diagnostic performance of PET/CT for malignant tumors. However, since the number of cases undergoing both 2-hour delayed imaging and PET/MR in this study was small, the sample size is relatively limited. Future studies should aim to increase the sample size to reduce experimental bias.

These findings also suggest that the conventional ^18^F-FDG metabolic tracer may not have significant imaging advantages for liver tumors (especially primary liver cancer and some metastases). In some well-differentiated primary liver cancers, tumor cells contain abundant glucose-6-phosphatase, which can dephosphorylate ^18^F-FDG-6-phosphate, converting it back to ^18^F-FDG, which is then transported out of the cells, making the tumor cells’ metabolism comparable to that of surrounding normal liver tissue. Despite the significant improvement in system resolution with Total-body PET/CT, this inherent limitation of the tracer itself also leads to false negatives (nine false-negative cases in this study). The new tracer ^11^C-acetate, involved in lipid metabolism, can reflect the biological behavior of tumors from another perspective. Domestic and international studies (18)have found that well-differentiated primary liver cancer has a high uptake of ^11^C-acetate. Therefore, new tracers, as important supplements to ^18^F-FDG, will significantly reduce false-negative results.

This study found the following: First, Total-body PET/CT improves system resolution and time resolution by increasing the axial field of view of PET/CT, thus enhancing the signal-to-noise ratio (SNR) and tissue-to-background ratio (T/B). This compensates for the limited soft tissue resolution of CT, but PET/MR still outperforms Total-body PET/CT in lesion detection rates due to its multiparametric, functional, metabolic, and anatomical imaging capabilities, as well as its superior soft tissue resolution. Second, although PET/MR significantly improves diagnostic performance for liver metastases, its high cost and low availability have limited its clinical use. In certain cases, 2-hour delayed Total-body PET/CT can compensate for the limitations of PET/CT and improve diagnostic accuracy for liver metastases.

The limitations of this study include: First, the sample size was relatively small, which may have introduced bias. Future research should focus on accumulating more cases and conducting large-scale prospective studies. Second, there are considerable anatomical and metabolic differences among liver metastases from various primary tumors. Future studies should expand the sample size and conduct subgroup analyses of different tumors using Total-body PET/CT and PET/MR. Third, PET/MR offers multiparametric imaging, particularly semi-quantitative parameters like DWI, SWI, and PWI. Future research should explore the correlation between these parameters and PET metabolic data to obtain more valuable diagnostic results (19, 20).

Currently, there is increasing interest in the application of imaging technologies for evaluating treatment response in tumors (21). The roles of PET/CT and PET/MR in assessing treatment efficacy in lymphoma are irreplaceable and future studies should focus on comparing the efficacy and prognosis of Total-body PET/CT and PET/MR in liver tumors (22, 23). Since ^18^F-FDG has limited utility as a metabolic tracer for liver tumors (especially primary liver cancer), and other malignant liver tumors have been relatively under-explored in imaging research (24), future research should focus on multiprobe, multi-histological imaging (25). We look forward to more optimized diagnostic methods and more clinical research to provide better opportunities for the early detection of liver metastases and to improve patient outcomes.

## Data Availability

The raw data supporting the conclusions of this article will be made available by the authors, without undue reservation.

## References

[1] Nuclear Medicine Committee Chinese Society of Clinical Oncology. Chinese expert cosensus on selective internal radiation therapy with yttrium-90 for primary and metastic hepatocellular carcinoma. Chin J Hepatol. (2021) 29:648–58. doi: 10.3760/cma.j.cn501113-20210302-00103 PMC12814040 34371535

[2] Asman Y Evenson AR Even-Sapir E Shibolet O. Fludeoxyglucose positron emission tomography and computed tomography as a prognostic tool before liver transplantation, resection, and loco-ablative therapies for hepatocellular carcinoma. Liver Transplant. (2015) 21:572–80. doi: 10.1002/lt.24083 25644857

[3] Boanova LG Altmayer S Watte G Raupp AA Francisco MZ De Oliveira GS. Detection of liver lesions in colorectal cancer patients using 18F-FDG PET/CT dual-time-point scan imaging. Cancers (Basel). (2023) 15. doi: 10.3390/cancers15225403 PMC10670707 38001662

[4] Zhou N Meng X Zhang Y Yu B Yuan J Yu J. Diagnostic value of delayed PET/MR in liver metastasis in comparison with PET/CT. Front Oncol. (2021) 11:717687. doi: 10.3389/fonc.2021.717687 34527587 PMC8435726

[5] Heusch P Nensa F Schaarschmidt B Sivanesapillai R Beiderwellen K Gomez B. Diagnostic accuracy of whole-body PET/MRI and whole-body PET/CT for TNM staging in oncology. Eur J Nucl Med Mol Imaging. (2015) 42:42–8. doi: 10.1007/s00259-014-2885-5 25112399

[6] Huellner MW Appenzeller P Kuhn FP Husmann L Pietsch CM Burger IA. Whole-body non-enhanced PET/MR versus PET/CT in the staging and restaging of cancers: preliminary observations. Radiology. (2014) 273:859–69. doi: 10.1148/radiol.14140090 25102372

[7] Zhang C O’Shea A Parente CA Amorim BJ Caravan P Ferrone CR. Evaluation of the diagnostic performance of positron emission tomography/magnetic resonance for the diagnosis of liver metastases. Invest Radiol. (2021) 56:621–8. doi: 10.1097/RLI.0000000000000782 33813576

[8] Grant AM Deller TW Khalighi MM Maramraju SH Delso G Levin CS. NEMA NU2–2012 performance studies for the SiPM-based TOF-PET component of the GE SIGNA PET/MR system. Med Phys. (2016) 43:2334–43. doi: 10.1118/1.4945416 27147345

[9] Wagatsuma K Miwa K Sakata M Oda K Ono H Kameyama M. Comparison between newgeneration SiPM-based and conventional PMT-based TOF-PET/CT. Phys Med. (2017) 42:203–10. doi: 10.1016/j.ejmp.2017.09.124 29173917

[10] Galgano S Viets Z Fowler K Gore L Thomas JV McNamara M. Practical considerations for clinical PET/MR imaging. Pet Clin. (2018) 13:97–112. doi: 10.1016/j.cpet.2017.09.002 29157390

[11] Cherry SR Jones T Karp JS Qi J Moses WW Badawi RD. Total-body PET: maximizing sensitivity to create new opportunities for clinical research and patient care. J Nucl Med. (2018) 59:3–12. doi: 10.2967/jnumed.116.184028 28935835 PMC5750522

[12] Xu BX Fu LP Guan ZW Yin DY Liu JJ Yang H. Comparison between PET/MR and PET/CT in evaluation of oncological patients. Chin J Nucl Med Mol Imaging. (2014) 34:423–7. doi: 10.3760/cma.j.issn.2095-2848.2014.06.002

[13] Lee DH Lee JM Hur BY Joo I Yi NJ Suh KS. Colorectal cancer liver metastases: diagnostic performance and prognostic value of PET/MR imaging. Radiology. (2016) 280:782–92. doi: 10.1148/radiol.2016151975 27092659

[14] Melsaether AN Raad RA Pujara AC Ponzo FD Pysarenko KM Jhaveri K. Comparison of whole-body F-18 FDG PET/MR imaging and whole-body F-18 FDG PET/CT in terms of lesion detection and radiation dose in patients with breast cancer. Radiology. (2016) 281:193–202. doi: 10.1148/radiol.2016151155 27023002 PMC5028256

[15] Schreiter NF Nogami M Steffen I Pape UF Hamm B Brenner W. Evaluation of the potential of PET-MRI fusion for detection of liver metastases in patients with neuroendocrine tumours. Eur Radiol. (2012) 22:458–67. doi: 10.1007/s00330-011-2266-4 21904802

[16] Lyu Q Lin D Tang M Liu D Zhang J Wang Y. 18F-FDG PET/CT and MR imaging features of liver metastases in gastrointestinal stromal tumors: a cross-sectional analysis. Ann Transl Med. (2022) 10:1220. doi: 10.21037/atm-22-5181 36544642 PMC9761173

[17] Beiderwellen K Geraldo L Ruhlmann V Heusch P Gomez B Nensa F. Accuracy of [18F]FDG PET/MRI for the detection of liver metastases. PloS One. (2015) 10:e0137285. doi: 10.1371/journal.pone.0137285 26335246 PMC4559465

[18] Zhao S Zhao L Zhang R Sun H. Application of ^11^C-acetate PET/CT combined with 18F-FDG PET/CT imaging in the diagnosis of primary liver cancer. Chin J Nucl Med Mol Imaging. (2018) 38:623–4. doi: 10.3760/cma.j.issn.2095-2848.2018.09.010

[19] Kickingereder P Wiesfler B Sahm F Heiland S Roethke M Schlemmer HP. Primary central nervous system lymphoma and atypical glioblastoma: multiparametric differentiation by using diffusion-, perfusion-, and susceptibility-weighted MR imaging. Radiology. (2014) 272:843–50. doi: 10.1148/radiol.14132740 24814181

[20] Tanyeri A Akbulut R Nevai EH Yürekli Y. Correlation of 3T diffusion-weighted MRI and 18F-FDG-PET/CT in liver metastases: SUV versus ADC. Mol Imaging Radionuc. (2025) 34:48–54. doi: 10.4274/mirt.galenos.2024.37431 PMC11827527 39918041

[21] Sun WJ Gao ZL Gao YJ Qiu HJ He YP Wang YJ. Quantitative evaluation of early stage blood flow change status after radiofrequency ablation based on multi-slice spiral CT whole-liver perfusion imaging on small hepatocellular carcinoma. Chin J Hepatol. (2020) 28:488–93. doi: 10.3760/cma.j.cn501113-20200317-00120 PMC12770228 32660177

[22] Ding CY Guo Z Sun J Yang WP Li TR. Prognostic value of pretreatment 18F-FDG PET-CT for patients with advanced diffuse large B-cell lymphoma. Chin J Oncol. (2018) 40:528–33. doi: 10.3760/cma.j.issn.0253-3766.2018.07.009 30060362

[23] He HH Wu XH Du XQ Mi BM Chen LP Zhang Y. Prognostic value of Deauville criteria and IHP criteria in 18F-FDG PET/CT for clinical evaluation at the end of treatment in diffuse large B-cell lymphoma. Chin J Nucl Med Mol Imaging. (2019) 39:266–71. doi: 10.3760/cma.j.issn.2095-2848.2019.05.003

[24] Li XS Zhang MC Qu YC Zhang XQ Pan F Liu YX. Diagnostic imaging of primary hepatic neuroendocrine tumors and treatment with transarterial chemoembolization: analysis of 6 cases. Chin J Hepatol. (2018) 26:294–7. doi: 10.3760/cma.j.issn.1007-3418.2018.04.012 PMC12769230 29996342

[25] Zhang MY Jiang H Zhang RJ Wang ZC Jiang HJ. 18F-FMISO PET evaluation of liver metastasis from colorectal cancer in mice. Imaging Diagnosis Interventional Radiol. (2020) 29:15–21. doi: 10.3760/cma.j.issn.1007-3418.2018.04.012

